# A Familial Form of Epidermolysis Bullosa Simplex Associated with a Pathogenic Variant in *KRT5*

**DOI:** 10.3390/genes12101503

**Published:** 2021-09-25

**Authors:** Francesco Paduano, Emma Colao, Teresa Grillone, Marco Flavio Michele Vismara, Rosario Amato, Steven Nisticò, Chiara Mignogna, Stefano Dastoli, Fernanda Fabiani, Rossella Zucco, Francesco Trapasso, Nicola Perrotti, Rodolfo Iuliano

**Affiliations:** 1 Medical Genetics Unit, Mater Domini University Hospital, 88100 Catanzaro, Italy; colaoemma@gmail.com (E.C.); teresagrillone@unicz.it (T.G.); marco.vismara@uniroma1.it (M.F.M.V.); rosario.amato@unicz.it (R.A.); fernandafabiani@libero.it (F.F.); trapasso@unicz.it (F.T.); perrotti@unicz.it (N.P.); 2 Department of Health Sciences, Campus S. Venuta, University Magna Graecia of Catanzaro, 88100 Catanzaro, Italy; nistico@unicz.it (S.N.); mignogna@unicz.it (C.M.); stefanodastoli@tiscali.it (S.D.); rossella.zucco@unicz.it (R.Z.); 3 Tecnologica Research Institute and Marrelli Health, Biomedical Section, Stem Cells and Medical Genetics Units, 88900 Crotone, Italy; 4 Department of Experimental and Clinical Medicine, Campus S. Venuta, University Magna Graecia of Catanzaro, Viale Europa, 88100 Catanzaro, Italy

**Keywords:** genodermatoses, EBS, *KRT5*, *KRT14*, cytokeratin

## Abstract

Epidermolysis bullosa simplex is a disease that belongs to a group of genodermatoses characterised by the formation of superficial bullous lesions caused by minor mechanical trauma to the skin. The skin fragility observed in the EBS is mainly caused by pathogenic variants in the *KRT5* and *KRT14* genes that compromise the mechanical stability of epithelial cells. By performing DNA sequencing in a female patient with EBS, we found the pathogenic variant c.967G>A (p.Val323Met) in the *KRT5* gene. This variant co-segregated with EBS in the family pedigree and was transmitted in an autosomal dominant inheritance manner. This is the first report showing a familial form of EBS due to this pathogenic variant.

## 1. Introduction

Hereditary epidermolysis bullosa (EB) is a heterogeneous group of rare genodermatoses, clinically characterised by skin and mucosal blistering that occur spontaneously or secondary to frictional and mechanical trauma [1]. EB was traditionally grouped in four major categories according to the region of blister formation in the epidermis, including Kindler syndrome, dystrophic EB, junctional EB, and EB Simplex (EBS) [1,2,3]. In addition, EB is subdivided into more than 39 subtypes based on ultrastructural features, clinical presentation, specific genetic variations, and immunohistochemical findings [4].

EB simplex (EBS) is the most common subtype of EB characterised by the fragility of the basal keratinocytes of the epidermis [5]. This weakness leads to bullous lesions of skin due to mechanical stress. The last updated recommendations on diagnosis and classification of EB were published in 2020 by Has and colleagues [6]. This novel reclassification system is not only clinically oriented but also considers the basis of the molecular defects found in patients with EBS [6]. Therefore, both specific clinical features and molecular findings should be considered for the correct classification of EBS. According to this recent reclassification, numerous clinical variants of EBS have been observed, including localised EBS (previously known as Weber-Cockayne), intermediate EBS (previously known as generalised intermediate or Koebner), and severe EBS (previously known as EBS Dowling-Meara) [6].

EBS is mainly caused by the pathogenic variants keratin 5 (*KRT5*) and 14 (*KRT14*) genes, which encode for the type I and II intermediate filaments expressed in the basal keratinocytes of the epidermis [7,8]. In their normal state, keratin 5 and keratin 14 form the intermediate keratin filament network that is directly linked to the desmosomes and hemidesmosomes, which are important for cell–cell and cell–basal lamina attachments, respectively [3]. Pathogenic variants in *KRT5* and *KRT14* genes can cause skin fragility due to the inability of keratinocytes to preserve their structural integrity during minor mechanical trauma on the skin with resulting skin blister formation [3]. Therefore, pathogenic variants in the basal keratinocyte-specific genes *KRT5* and *KRT14* cause the basal keratinocytes of the epidermis to become fragile and subjected to rupture upon skin trauma.

Hereditary EBS associated with pathogenic variants in *KRT5* or *KRT14* is predominantly inherited in an autosomal dominant manner, but also an autosomal recessive inheritance has been observed in rare families [5,6].

Here, we reported the rare c.967G>A heterozygous *KRT5* pathogenic variant from a non-consanguineous south Italian family that had the typical clinical features of intermediate EBS.

To our knowledge, the pathogenic variant c.967G>A in the *KRT5* gene has been previously reported only in a sporadic case of EBS [9]; however, this is the first report showing a familial form of intermediate EBS due to this variant.

## 2. Materials and Methods

### 2.1. Patient and Family Members

**Figure 1:**
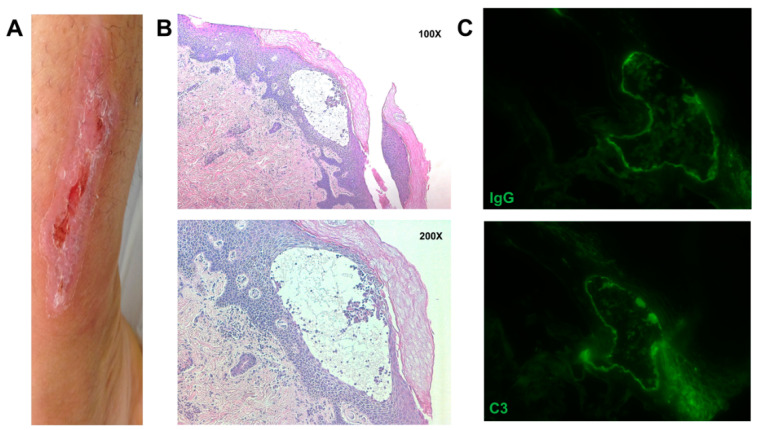
(**A**) Image showing the clinical skin manifestation of the patient with EBS. (**B**) IHC images showing intraepidermal blistering, mild spongiosis with associated inflammatory infiltrate (lymphoplasmacellular cells). Dermis shows mild inflammatory infiltrate of perivascular lymphocytes, magnification 100× and 200×. (**C**) Immunofluorescent analysis for C3 and IgG showing green signal at the dermo-epidermal junction, magnification 400×.

Here, we report the case of a 29-year-old woman with skin blistering and cutaneous lesions due to frictional trauma (Figure 1A), who accessed our medical genetics unit seeking genetic counselling for the inheritance of her condition. She presented clinical features of EBS soon after her birth. During infancy, she periodically suffered from bullae and skin blistering that increased in the summer season. Her condition ameliorated with advancing age, and now the skin manifestations occur mainly after mechanical stress episodes or intense physical activity. These clinical features led us to consider this phenotype as EBS- intermediate. She treats her condition with bland emollients and rupturing of blisters with sterile needles. In addition, when needed, she used topical antibiotics and an oral analgesic to relieve the pain. At the age of 18 years, the patient started to suffer axillary hidradenitis suppurativa. This condition recurred three times in three years, and it was then surgically treated. Since multiple components in the paternal branch of the patient were affected by intermediate EBS from birth, some of the patient’s relatives were recruited and visited our clinical unit. Physical examination showed a similar skin phenotype in the patient’s family pedigree: an episode of axillary hidradenitis suppurativa was observed in the patient’s father (II.1), whereas typical EBS skin lesions were observed in the left ankle of the patient’s male cousin (III.8) and the foot of patient’s uncle (II.9). The patient’s mother (II.2) and sister (III.2) did not present the typical clinical features of EBS.

EBS features, including hyperpigmentation, plantar keratoderma, EB nevi, eye, hair and nail involvement, were absent in the index patient as well as in family members.

### 2.2. Immunohistochemistry (IHC) and Immunofluorescence (IF)

The skin samples were obtained from the skin around the recent blister. IHC staining was performed on biopsy samples as previously described [10]. Samples for IF were stained using FITC-conjugated polyclonal rabbit antisera against human IgG and C3. The immune deposits were observed under a fluorescence microscope.

### 2.3. Molecular Analysis

Genomic DNA was isolated from peripheral blood using a Blood DNA Extraction Kit as previously described [11,12]. The selected exons of *KRT5* (1, 2, 5, 7, and 9) and *KRT14* (4, 5, 6, and 7) were amplified and checked by sequencing analysis (ABI Prism 310 Genetic Analyzer) as previously described [13]. NM_000526.3 and NM_000424.3 were used as reference sequences for *KRT14* and *KRT5,* respectively.

### 2.4. Statement of Ethics

Written informed consent was taken for the images present in this case report. The authors declare no ethical conflicts to disclose. All genetic tests for the patient and patient’s family members were performed after their informed consent in accordance with the Helsinki Declaration and the approval of the local Ethics Committee.

## 3. Results

The histological evaluation of the patient’s skin biopsy showed an intradermal tissue separation with degeneration of the basal cell layer (Figure 1B). Surprisingly, the pathologist also found IgG and C3 deposition that were compatible with the epidermolysis bullosa acquisita (EBA) (Figure 1C). It is possible that the tissue damage secondary to the genetic mutation triggers an autoinflammatory reaction that may explain this peculiar histologic phenotype [14].

**Figure 2:**
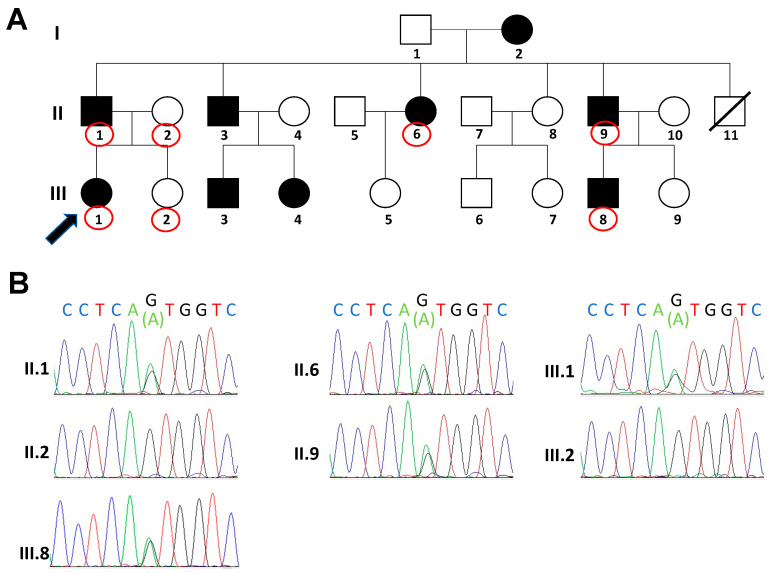
(**A**) Family pedigree of the patient and her relatives. Affected individuals are shown as filled symbols, whereas the patient is identified by the arrow. Family members analysed for *KRT5* pathogenic variant are indicated by red circles. (**B**) Sequence electropherograms of *KRT5* exon 5 region containing the G > A transition (c.967 G > A) (pVal323Met). The pathogenic variant is present in affected family members only.

Since a clinical diagnosis of intermediate EBS (previously known as generalised intermediate or Koebner) was made for the patient (III.1, Figure 2A), we first considered just exons 1, 2, 5, 7 and 9 of *KRT5* and exons 4, 5, 6 and 7 of *KRT14* to look for pathogenic variants associated with intermediate EBS (Human Intermediate Filament Database, www.interfil.org, accessed on 1 July 2021) [15]. Sanger sequencing showed that the patient (III.1) was heterozygous for the pathogenic variant ([GRCh37/hg19] chr12: g.52911499C > T; NM_000424.3: c.967G > A (pVal323Met) in exon 5 of *KRT5* gene (III, 1. Figure 2B). No genetic alterations were detected within the *KRT14* gene.

The patient’s family members II.1, II.6, II.9 and III.8, who had clinical manifestations typical of intermediate EBS, were all heterozygous carriers of the c.967G>A *KRT5* variant, whereas the patient’s family members II.2 and III.2, who were healthy, were found to have a wild-type *KRT5* allele (Figure 2B). Notably, the pathogenic variant was present only in affected family members, and the family pedigree was compatible with an autosomal dominant inheritance. The *KRT5* pathogenic variant detected here was not found in the GnomAD database [16]. In addition, the PredictSNP used to evaluate the possible pathogenicity of this variant predicted that the c.967G > A *KRT5* variant is deleterious (Table 1) [17].

The *KRT5* heterozygous variant described here, not previously described in any database including ClinVar, LOVD and HGMD, was classified as likely pathogenic based on the American College of Medical Genetics (ACMG) criteria (PM1 + PM2 + PM5 + PP2 + PP3) [18].

**Table 1:** PredictSNP analysis of pathogenic variants detected at the amino acid 323 of KRT5.

Pathogenic Variant	Predicted Phenotype	Expected Accuracy	EBS Type	Reference
V323A	Deleterious	61%	Generalised Localised	[19,20]
V323G	Deleterious	72%	Localised	[21]
V323M	Deleterious	76%	Generalised	Our study

## 4. Discussion

Here, we present a family that had five subjects affected by intermediate EBS (previously known as generalised intermediate or Koebner). In generalised EBS, the formation of the blisters begins at birth or develops in the first few months of life. The sites of predilection are feet, hands, knees, elbows and legs, but it can also affect the whole body; other clinical features are nail dystrophy, milia, palmar and plantar hyperkeratosis [7]. Currently, the identification of pathogenic variants in *KRT5* or *KRT14* by molecular genetic testing and confirmation by histological examination and immunofluorescence antigen mapping (IFM) are used for the diagnosis of EBS [19].

The product of the *KRT5* gene is the cytokeratin 5, a protein of 58 kDa that belongs to the type II cytokeratin family. It is specifically expressed in the basal layer of the epidermis with type I cytokeratin member *KRT14*. It possesses an N-terminal DNA-binding domain (167 amino acids), containing a Gly-rich motif, a central rod region spanning from position 168 to 477, and a C-terminal tail. The last 62 residues form a serine-rich motif [21].

The rod region is further divided into three coil regions that are separated by two linker regions. The pathogenic variant reported here lies in the 323rd residue, in the second linker region, 23 amino acids before the third coil region, named coil 2 (the first two are called coil 1a and coil 1b, respectively). Coil 2 spans from position 339 to 477 and is prevalently occupied by the coiled-coil region, which dimerises with cytokeratin 14.

Pathogenic variants of *KRT5* lie prevalently in coil 1a, in the second linker region and the terminal part of coil 2. Pathogenic variants in the linker regions are commonly associated with milder phenotypes. Conversely, severe EBS diseases have pathogenic variants in the helix motifs of *KRT5* and *KRT14* [20,22,23]. Most pathogenic variants positioned in linker 12 region are associated with localised EBS, although changes in amino acids at position 323 and 331 can lead to the more severe generalised EBS [21,22,24]. In this context, Valine 323 appears to be a relevant residue in the L12 region, enforced by the high conservation of this amino acid among species and in the intermediate filament proteins [19].

The majority of *KRT5* pathogenic variants that cause EBS act in a dominant manner. In our cases of intermediate EBS, we found a missense pathogenic variant (valine to methionine) at position 323 of *KRT5*. This pathogenic variant has been previously detected in a sporadic case of Koebner-type EBS [9]. Different from the reported sporadic case indicated above, oral mucosa was never affected in our patients, suggesting that the genomic context is essential to determine the severity of the disease. Other pathogenic variants, Val323Gly and Val323Ala, at the same amino acid residue of KRT5, were associated with EBS, confirming that Valine 323 (V323) is an essential residue for KRT5 conformation [19,21].

Pathogenic variants lying in the linker regions are often associated with the mildest EBS phenotype unless the residue involved is well conserved among species, as in our case. When this occurs, a more severe phenotype can be expected.

Importantly, we found an intriguing association between EBS and hidradenitis suppurativa (HS). In fact, the proband and her father also suffered from HS. The association of EBS with hidradenitis suppurativa has been observed in some patients [25]. HS is a chronic inflammatory skin disease that involves innate inflammatory cytokines and cells linked to inflammatory pathways. TH17 cells play an important role in the pathogenesis of HS, and the activation of these cells has also been related to EBS with *KRT5* mutations [26,27]. As a matter of fact, proinflammatory cytokines and anti-skin antibodies have been detected in patients with hereditary EBS [28], and autoimmunity has been interpreted as both a consequence and cause of further skin deterioration in hereditary EB due to a state of chronic inflammation [14].
